# Personality Traits and Disorders in Adolescents at Clinical High Risk for Psychosis: Toward a Clinically Meaningful Diagnosis

**DOI:** 10.3389/fpsyt.2020.562835

**Published:** 2020-12-08

**Authors:** Tommaso Boldrini, Annalisa Tanzilli, Giuseppe Di Cicilia, Ivan Gualco, Vittorio Lingiardi, Silvia Salcuni, Maria Cristina Tata, Stefano Vicari, Maria Pontillo

**Affiliations:** ^1^Department of Developmental Psychology and Socialization, University of Padua, Padua, Italy; ^2^Department of Dynamic and Clinical Psychology, Sapienza University of Rome, Rome, Italy; ^3^Center for Individual and Couple Therapy, Genoa, Italy; ^4^Child and Adolescence Neuropsychiatry Unit, Department of Neuroscience, Children Hospital Bambino Gesú, IRCCS, Rome, Italy; ^5^Department of Life Sciences and Public Health, Catholic University, Rome, Italy

**Keywords:** clinical high risk (CHR) for psychosis, personality, adolescence, early detection & prevention, personality traits

## Abstract

**Aims:** Recent meta-analytic data show that approximately 40% of individuals at clinical high risk for psychosis (CHR) receive at least one personality disorder (PD) diagnosis. Personality pathology could significantly influence CHR patients' prognosis and response to treatment. We aimed at exploring the PD traits of CHR adolescents, in order to outline a prototypic description of their most frequently observed personality characteristics.

**Methods:** One hundred and twenty-three psychiatrists and psychologists used a Q-sort procedure [i.e., the Shedler–Westen Assessment Procedure-200 for Adolescents (SWAP-200-A)] to assess personality traits and disorders in 58 (30 male; mean age = 16 years, range = 13–19 years) CHR adolescents and two gender- and age-matched samples, respectively, with (*n* = 60) and without PDs (*n* = 59).

**Results:** Differences between the CHR, PD, and clinical groups showed that CHR adolescents had pervasive and more clinically relevant schizoid, schizotypal, borderline, and avoidant traits, as well as poorer adaptive functioning. Moreover, by collecting the highest mean SWAP-200-A items, we empirically outlined a prototypic description of CHR youths, comprised of avoidance of social relationships; suspiciousness; obsessional thoughts; lack of psychological insight; dysphoric and overwhelming feelings of anxiety and depression; odd and anomalous reasoning processes or perceptual experiences; symptoms of depersonalization and derealization; and negative symptoms of avolition, abulia, blunted affects, and impaired role functioning.

**Conclusions:** The results suggest that avoidant interpersonal strategies, impaired mentalization, and difficulties in emotional regulation could become important targets for psychosocial interventions with CHR adolescent populations.

## Introduction

Over the last two decades, two complementary sets of operational criteria have been developed to identify young people putatively considered at imminent risk for developing a psychosis spectrum disorder (1). First, the ultra-high risk (UHR) criteria refer to attenuated psychotic symptoms (APS), brief limited intermittent psychotic symptoms (BLIPS), and genetic vulnerability associated with a marked decline in psychosocial functioning [genetic risk and deterioration syndrome (GRD)] (2). Second, the basic symptoms (BS) criteria describe subjectively experienced subclinical disturbances in perception, thought processing, language, and attention; such symptoms are phenomenologically distinct from those of full-blown psychosis, as the patient's insight and reality testing are preserved (3, 4). Longitudinal research has suggested that individuals at clinical high risk for psychosis (CHR; i.e., individuals meeting UHR and/or BS criteria) are up to 20 times more likely to develop psychosis, compared to the general population (5).

Evidence has revealed that the CHR population may display heterogeneous clinical presentations and a high prevalence of psychiatric syndromes—particularly depressive and anxiety disorders—which may influence the psychopathological frame and treatment outcome (6–8). Moreover, reports from the largest studies in the field—such as the Prevention through Risk Identification, Management, and Education [PRIME (9)] and the Recognition and Prevention [RAP (10)] programs, as well as the North American Prodrome Longitudinal Study [NAPLS (11)]—have shown that certain personality disorders (PDs) are prevalent among CHR adolescents and young adults. Indeed, a recent and comprehensive meta-analysis (12) of 17 empirical investigations (*n* = 1,868) showed a 39.4% prevalence rate of PDs (at least one PD diagnosis) within this population. In particular, 13.4 and 11.9% of the CHR patients suffered from schizotypal and borderline PDs, respectively. These rates are four times larger than those of the general population (13) and roughly equivalent to those reported in previous meta-analyses concerning other clinical psychiatric diagnoses [e.g., 41% for depressive disorders and 34.4% for anxiety disorders (2, 4, 5)].

Despite the high prevalence and variability of PDs among CHR individuals (12), studies on the psychosis-predictive value of PDs have generated mixed results, highlighting a potential impact of schizoid and borderline PDs only (12, 14)^1^. However, PD diagnoses might contribute to explaining the severe distress and disability of CHR patients, difficulties in their provision of care, and differences in their responses to treatment (12, 16).

Overall, we propose that studies of personality features in CHR research have suffered from at least one major limitation, linked to their assessment procedures. In fact, the great majority of studies in this field (16–19) have used self-report measures or structured interviews to assess personality pathology in CHR patients [e.g., the Structured Clinical Interview for *Diagnostic and Statistical Manual of Mental Disorders 4th ed*. [SCID (20)]; the Millon Multiaxial Inventory, Version III [MCMI-III (21)]]. Such instruments may suffer from several weaknesses. For example, many personality features cannot be measured via direct questioning, due to the implicit nature of their underlying cognitive and affective processes and/or respondents' lack of self-awareness or defensive biases (e.g., respondents may provide misleading information when describing socially undesirable symptoms or traits) (22, 23).

Such limitations may be especially pronounced in research involving patients with a schizophrenia-spectrum disorder. Boberg et al. (24) showed that outcomes from the Structured Clinical Interview for *Diagnostic and Statistical Manual of Mental Disorders 5th ed*. (SCID-5) (25) were only marginally correlated with the diagnoses of expert clinicians. In particular, when considered alone (i.e., without clinician assessments), the interview misdiagnosed a high proportion of schizophrenia-spectrum disorders as PDs (in particular, borderline PDs) and tended to overlook schizotypal PDs. Clinician-report methods for assessing personality rely on the observations of experienced raters and their longitudinal knowledge of patients. For this reason, such measures can overcome the abovementioned biases, ensuring greater validity (26).

Starting from these premises, the present study aimed at deepening our understanding of personality traits and disorders in the CHR adolescent population. An accurate assessment of patients' features could have relevant clinical implications, particularly in promoting patient-tailored interventions to enhance treatment effectiveness (26). In the study, we asked a sample of experienced clinicians to describe their patients (with a positive or negative CHR status) by rating 200 descriptors (items) on a Q-sort assessment tool (i.e., the Shedler–Westen Assessment Procedure for Adolescents [SWAP-200-A (27, 28)]) pertaining to a wide range of personality and clinical characteristics. The SWAP-200 (see also “Measures” section) was designed to provide a comprehensive assessment of patients' personality and psychological functioning by quantifying clinical observations. The use of this assessment procedure enabled us to address the relevant methodological shortcomings of previous studies in the field.

In more detail, we investigated personality traits and personality pathology in a group of CHR individuals, in comparison with two adolescent clinical groups of non-CHR subjects, respectively, with and without a PD diagnosis. Second, we aimed at producing an empirically derived prototypic description of personality characteristics in the CHR population, in terms of affective states and emotional regulation strategies; interpersonal functioning; cognitive styles; mental representations of self, others, and the interaction between self and others; and overall psychological functioning.

## Method

### Participant Sampling

Three clinical populations of outpatients were recruited from Italian National Health System centers and public associations providing psychotherapeutic treatment to adolescent and young adult patients with a CHR condition or different psychopathological presentation. Specifically, data were collected from: (a) a sample of CHR patients enrolled at the Child and Adolescent Neuropsychiatry Unit of the Bambino Gesù Pediatric Hospital in Rome and (b) two distinct samples of patients, respectively, with or without a PD, who were enrolled in psychotherapy associations in Genoa, Milan, Rome, and Turin.

Inclusion criteria for all participants were: (a) aged 13–19 years; (b) no psychotic psychiatric disorder based on the *DSM-5* (25) classification system; (c) no traumatic brain injury, neurological disorder, or clinically significant cognitive impairment; (d) fluency in Italian; and (e) IQ > 70.

Clinicians from the Bambino Gesù Pediatric Hospital were asked to select patients who satisfied at least one UHR criterion (29), such as APS, brief intermittent psychotic syndrome (BIPS), and/or GRD, with no full-blown psychotic disorder and/or a Presence of Psychotic Symptoms (POPS) state according to the Structured Interview for Prodromal Syndromes (SIPS) (see “Measures” section). Conversely, clinicians from other recruitment sites were asked to select non-CHR patients, in accordance with the following exclusion criteria: (a) no clinical presentations referable to the psychosis spectrum, including the *DSM-5* attenuated psychosis syndrome (25), which has recently been shown to have significant concurrent and prognostic validity (30); (b) no predominantly psychotic disorders (especially, no condition related to the prodromal phase of schizophrenia), according to the Psychodiagnostic Chart [PDC-A; (31)]; and (c) no high scores (>3) on subscales relevant to psychosis (i.e., Paranoid Ideation, Psychoticism) on the Symptom-Checklist 90–Revised (SCL-90-R) (32). All participants were drug-naïve patients at the time of the first clinical interview.

Research data on the patients who met the abovementioned criteria were provided by a wide group of clinicians (clinical psychologists and psychiatrists), who were asked to conduct a comprehensive diagnostic assessment of their patients' personality and psychological functioning.

The study obtained approval from the Ethics Committee of the Bambino Gesù Pediatric Hospital and the Ethics Committee of the Department of Dynamic and Clinical Psychology, Sapienza University of Rome (n°44/2017). All clinicians furnished written informed consent and were instructed to withhold any identifying information about their patients. They received no remuneration for their participation. Adolescent patients were not directly involved in this study.

### Practitioners

The sample consisted of 123 clinicians: 76 female (62%) and 47 male (38%). The mean age of all practitioners was 45.15 years (*SD* = 7.82, range = 27–61). Twenty-five (20%) were psychiatrists, and 98 (80%) were clinical psychologists. The average length of their clinical experience was approximately 12 years (*SD* = 7.53, range = 2–31). All clinicians received the same formal training for the SWAP-200-A (see “Measures” section)—provided by two authors of the present paper—and obtained an IRR in the range of 0.69–0.75 when assessing video-recorded therapy sessions with different patients. All SWAP-200-A assessments were performed after patients had participated in at least five psychotherapy sessions, to ensure that clinicians had deep and longitudinal knowledge of their patients. Specifically, the mean number of psychotherapy sessions provided by clinicians to each patient before the SWAP-200-A assessment was 8.63 (SD = 1.2; range = 5–12).

### Patients

The population examined in the present study consisted of 177 individuals, subdivided into the following samples.

#### Clinical High Risk (CHR) for Psychosis Group

This group consisted of 58 help-seeking inpatients (30 female, 28 male) who had been consecutively admitted to the Child and Adolescent Neuropsychiatry Unit of the Bambino Gesù Pediatric Hospital in Rome between January 2017 and October 2019. Their mean age was approximately 16 years (*SD* = 1.6; range = 13–19). All patients who met the eligibility criteria were approached, and the majority agreed to participate (response rate, 78%). Most patients (62%) presented at least one comorbid clinical diagnosis. In particular, 14 were diagnosed with a generalized anxiety disorder, 10 with a panic disorder, 6 with a persistent depressive disorder (dysthymia), and 6 with a major depressive disorder. Notably, many patients had been referred to the Bambino Gesù Pediatric Hospital by other psychiatric clinicians, on the suspicion that they were at risk for developing psychosis. This resulted in a “pre-assessment enrichment,” which conferred great validity of the UHR criteria (33, 34).

#### Personality Disorder (PD) Group

This group consisted of 60 patients (30 female, 30 male) who had been diagnosed with a PD according to the *DSM-5* classification system. Their mean age was approximately 16 years (*SD* = 1.6; range = 13–18). Nine had a Cluster A diagnosis, 28 had a Cluster B diagnosis, and 23 had a Cluster C diagnosis.

#### Clinical Group

This group consisted of 59 patients (38 female, 21 male) who had been diagnosed with various clinical syndromes (without PD comorbidity), according to the psychopathological categories of the *DSM-5* classification system. Their mean age was 16 years (SD = 1.4; range = 13–18). The majority of these adolescents presented different syndromes, including anxiety, depressive, and feeding and eating disorders. In particular, 14 were diagnosed with a generalized anxiety disorder, 11 with a feeding and eating disorder, 10 with a panic disorder, 7 with a persistent depressive disorder (dysthymia), 6 with a major depressive disorder, 6 with an attention-deficit/hyperactivity disorder, and 5 with an oppositional defiant disorder.

### Measures

#### Clinical Questionnaire

We used a clinician-report questionnaire (35) to collect comparable general information about the different patient populations. Clinicians provided basic demographic data for patients, as well as patients' *DSM-5* diagnoses at intake. Moreover, the questionnaire gathered information on all clinicians (with respect to, sex, age, years of experience, and profession).

#### Shedler–Westen Assessment Procedure-200 for Adolescents [SWAP-200-A (27, 28)]

The SWAP-200-A is a clinician-report instrument for assessing personality pathology and psychological functioning in adolescent patients; it is used for both clinical and research purposes (36, 37). The measure was adapted from the SWAP-200 for adults (38, 39), and it comprises 200 statements written in jargon-free language, describing pathological and healthy features of adolescent personality. To describe a young patient using the SWAP-200-A Q-sort, an experienced clinician scores each of the 200 items on a scale ranging from 0 (*irrelevant or not descriptive*) to 7 (*highly descriptive*), according to a fixed distribution. A computer program then provides dimensional and categorical diagnoses for: (a) 10 PD prototypes (Paranoid, Schizoid, Schizotypal, Antisocial, Borderline, Histrionic, Narcissistic, Avoidant, Dependent, and Obsessive-Compulsive *PD scales*) and (b) 6 personality styles/disorders (Antisocial-Psychopathic, Emotional-Dysregulated, Histrionic, Narcissistic, Avoidant-Constricted, and Inhibited Self-Critical *Q-factors*). Final scores are presented as T-points, with scores in the range of 55–60 considered indicative of sub-threshold or mild pathology or PD and scores > 60 considered indicative of severe pathology or PD. These results enable a taxonomy of adolescent personality to be drawn (36). Moreover, the SWAP-200-A also considers high-functioning personality characteristics and includes an index of healthy personality functioning to detect clinically relevant strengths and resources. In this study, we used only the SWAP-200-A PDs and High-Functioning scales. The overall measure has been shown to have excellent psychometric properties (36).

#### Structured Interview for Prodromal Syndromes (SIPS)

The SIPS (40, 41) is a structured diagnostic interview comprised of four measures: (1) the Scale of Prodromal Symptoms (SOPS), (2) the *DSM-IV* Schizotypal Personality Disorder Checklist, (3) a questionnaire pertaining to family history of mental illness, and (4) the Global Assessment of Functioning scale. The SOPS assesses 19 symptom constructs across four subscales: Positive Symptoms (five items), Negative Symptoms (six items), Disorganization Symptoms (four items), and General Symptoms (four items). For each of these subscales, symptoms are rated on a seven-point Likert scale ranging from 0 (*never*) to 6 (*severe*). Scores of 3, 4, or 5 on at least one of the positive items are sufficient to meet the classification criteria for the CHR condition. Conversely, a score of 6 indicates the presence of a full-blown psychotic syndrome (POPS criteria). At the end of the evaluation procedure, the SIPS provides diagnostic criteria for three psychosis-risk syndromes: (1) BIPS; (2) attenuated positive symptom syndrome (APSS); and (3) GRD, characterized by schizotypal PD and/or first-degree familiarity with schizophrenia-spectrum disorders and a significant decline in global functioning over the past 12 months. The SIPS has been found to have excellent inter-rater reliability and predictive validity (41).

### Statistical Analysis

Statistical analyses were carried out using SPSS 20 for Windows (IBM, Armonk, NY). A χ^2^ analysis and an analysis of variance (ANOVA) were conducted to compare CHR, PD, and clinical adolescent groups on some demographic variables (sex and age). Group differences in patients' PDs and psychological functioning (evaluated using the SWAP-200-A) were analyzed using a multivariate analysis of variance (MANOVA) with Bonferroni *post hoc* analyses (*p* < 0.05). The MANOVA was conducted to examine the data at the individual disorder level, considering all SWAP-200-A PD scales. Finally, we composed an empirically derived prototype of CHR personality to identify the specific psychological features that characterize this adolescent population. For this purpose, SWAP-200-A items across CHR patients were standardized (z-scored), and item scores were averaged to create a composite personality profile.

## Results

### Sample Characteristics

The total sample was comprised of 177 participants: 98 female (55.37%) and 79 male (44.63%). The mean age of the sample was 16 years (*SD* = 1.52; range = 13–19). The three subsamples of CHR, PD, and clinical adolescents were compared on demographic variables (sex and age). The χ^2^ analysis did not reveal any significant difference between groups in terms of sex, χ^2^ = 2.96, *p* = 0.23. Similarly, no significant difference was found by the ANOVA in terms of age, *F*_(2, 174)_ = 0.13, *p* = 0.88, η^2^ = 0.01.

### Group Differences in Personality Pathology and Psychological Functioning

The first aim of the present study was to compare the CHR, PD, and clinical adolescent groups on PDs and global psychological functioning (assessed by the SWAP-200-A PD and High-Functioning scales). A MANOVA was conducted, using groups as the independent variable and all SWAP-200-A PD scales as dependent variables. The findings showed significant main effects for the groups on the SWAP-200-A PD and High-Functioning scales, Wilks's λ = 0.22, *F*_(22, 328)_ = 17.18, *p* < 0.001, η^2^ = 0.54 (Table 1).

**Table 1:** Differences between CHR, PD, and clinical adolescent groups on SWAP-200-A PDs and global psychological functioning (*N* = 177).

**SWAP-200-A PD scale**	**CHR group (*****n*** **=** **58)**		**PD group (*****n*** **=** **60)**		**Clinical group (*****n*** **=** **59)**			
	***M***	***SD***	***M***	***SD***	***M***	***SD***	***F*_**(2, 174)**_**	**η^**2**^**
Paranoid	43.42	0.46	43.60	0.45	42.20	0.45	2.83	0.03
Schizoid	51.55^a^	0.95	48.19^b^	0.93	46.39^c^	0.94	7.71^***^	0.08
Schizotypal	55.20^a^	0.91	49.14^b^	0.90	44.65^c^	0.90	34.11^***^	0.28
Antisocial	45.35^a^	0.92	51.17^b^	0.91	44.87^a^	0.92	14.86^***^	0.15
Borderline	46.66^a^	1.02	49.13^a^	1.00	43.08^b^	1.01	9.15^***^	0.10
Histrionic	46.81^a^	0.98	51.40^b^	0.96	46.61^a^	0.97	7.86^***^	0.08
Narcissistic	43.72^a^	1.05	49.84^b^	1.03	43.37^a^	0.1.04	8.69^***^	0.09
Avoidant	47.77^a^	0.87	48.64^a^	0.86	43.94^b^	0.86	8.40^***^	0.09
Dependent	45.80^a^	0.88	49.47^b^	0.87	45.70^a^	0.88	6.09^**^	0.07
Obsessive	42.94^a^	0.65	45.34^b^	0.64	41.93^a^	0.64	7.54^***^	0.08
High-functioning	47.71^a^	0.74	48.61^a^	0.73	55.20^b^	0.73	31.17^***^	0.26

*CHR group, clinical high-risk group; PD group, personality disorder group; SWAP-200-A, Shedler–Westen Assessment Procedure-200 for Adolescents; η^2^, measure of effect size in analysis of covariance. Alphabetical superscripts indicate significant differences in the post hoc analyses. Means with different alphabetic superscripts (a, b, and c) were statistically significant, while means with identical alphabetic superscripts were not significantly different*.

** *p < 0.01*.

*** *p < 0.001*.

**Figure 1:**
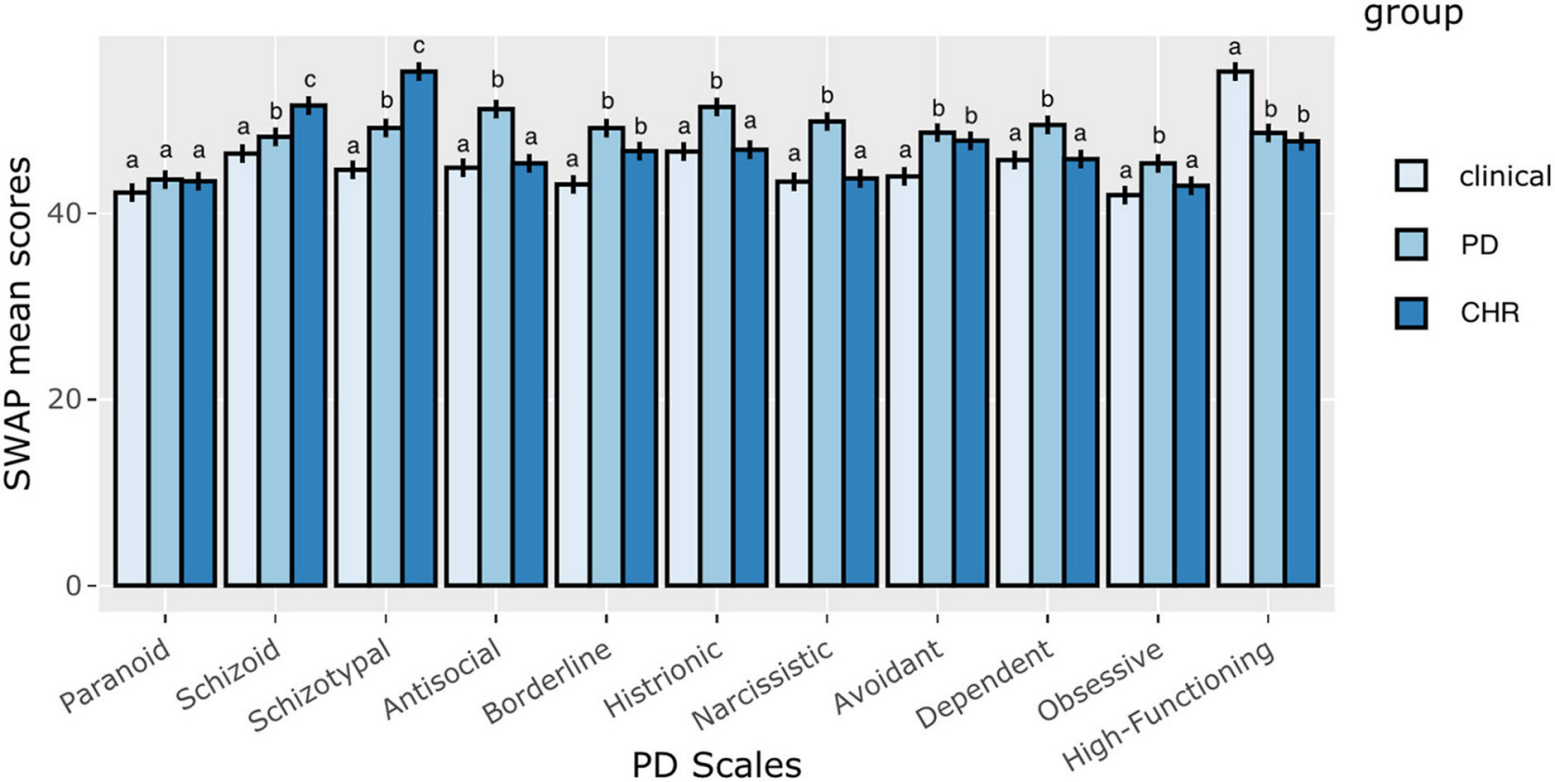
CHR group, clinical high-risk group; PD group, personality disorder group; SWAP-200-A, Shedler–Westen Assessment Procedure-200 for Adolescents. Alphabetical superscripts indicate significant differences in the *post hoc* analyses. Means with different alphabetic superscripts (a, b, and c) were statistically significant, while means with identical alphabetic superscripts were not significantly different.

The *post hoc* analyses using Bonferroni's correction showed significant differences between the CHR, PD, and clinical adolescent groups on all SWAP-200-A PD scales, except for the Paranoid scale (Figure 1). The CHR adolescent group had significantly higher mean scores in the SWAP-200-A Schizoid and Schizotypal PD scales than the PD and clinical groups. Moreover, the CHR and PD groups had significantly higher mean scores in the SWAP-200-A Borderline and Avoidant PD scales and lower mean scores in the SWAP-200-A High-Functioning scale than the clinical group. For the remaining SWAP-200-A PD scales (Antisocial, Histrionic, Narcissistic, Dependent, and Obsessive-Compulsive), the PD patient group had significantly higher mean scores than the CHR and clinical groups.

### Empirically Derived Prototype of CHR Personality

The second aim of this study was to provide an empirically derived prototype of the CHR personality, creating a composite description of the specific psychological traits that characterize these patients. Table 2 shows the SWAP-200-A items that obtained the highest mean scores and were most descriptive of personalities in the CHR sample. A multifaceted portrait was obtained, indicating a pattern of avoidance of interpersonal relationships (item 124), associated with feelings of shame, shyness, embarrassment, and fear of rejection (items 60, 98, 54); a tendency to express suspicion toward others (items 105, 87); obsessional thoughts (item 6); severely impaired mentalization, in both self-oriented (item 148) and other-oriented dimensions (item 29); emotional dysregulation (items 12, 117), with dysphoric feelings of anxiety (item 35) and depression (item 189); odd and anomalous reasoning or perceptual experiences (items 44, 130), especially when under stress; dissociative symptoms of depersonalization and derealization (item 138); and negative symptoms of avolition (item 30), abulia and blunted affects (item 119), and impaired role and academic/occupational functioning (item 188).

**Table 2:** SWAP-200-A items most descriptive of the personality and psychological functioning of CHR adolescent patients (*N* = 58).

**Empirically derived prototype**	
**20 most descriptive items of the SWAP-200-A**	**Mean**
35. Tends to feel anxious.	1.42
44. When distressed, perception of reality can become grossly impaired (e.g., thinking may seem delusional).	1.41
60. Tends to be shy or self-conscious in social situations.	1.18
189. Tends to feel unhappy, depressed, or despondent.	1.12
124. Tends to avoid, or try to avoid, social situations because of fear of embarrassment or humiliation.	1.11
188. Her/his psychological problems interfere with an adequate academic performance (or with an adequate working capacity, if s/he no longer goes to school).	1.08
130. Reasoning processes or perceptual experiences seem odd and idiosyncratic (e.g., may make seemingly arbitrary inferences; may see hidden messages or special meanings in ordinary events).	1.06
12. Emotions tend to spiral out of control, leading to extremes of anxiety, sadness, rage, etc.	1.02
138. Tends to enter altered, dissociated states when distressed (e.g., the self or world feels strange, unreal, or unfamiliar).	0.95
6. Is troubled by recurrent obsessional thoughts that s/he experiences as senseless and intrusive.	0.90
30. Tends to feel listless, fatigued or lacking in energy.	0.89
29. Has difficulty making sense of other people's behavior; often misunderstands, misinterprets, or is confused by others' actions and reactions.	0.84
54. Tends to feel s/he is inadequate, inferior, or a failure.	0.84
87. Is quick to assume that others wish to harm or take advantage of her/him; tends to perceive malevolent intentions in others' words and actions.	0.83
105. Is suspicious; tends to assume others will harm, deceive, conspire against, or betray her/him.	0.82
117. Is unable to soothe or comfort her/himself without the help of another person (i.e., has difficulty regulating own emotions).	0.78
148. Has little psychological insight into own motives, behavior, etc.	0.76
86. Tends to feel ashamed or embarrassed.	0.75
98. Tends to fear s/he will be rejected or abandoned by those who are emotionally significant.	0.71
119. Tends to be inhibited or constricted; has difficulty allowing self to acknowledge or express wishes and impulses.	0.69

## Discussion

The first aim of the present study was to examine differences between CHR, PD, and clinical groups pertaining to personality disorder traits. In line with previous studies (12), the results revealed that CHR patients had a higher prevalence of schizoid and schizotypal traits, compared to the other groups. Schizoid PDs have been rarely considered in CHR research, with the exception of a study by Shultze-Lutter et al. (16), which found schizoid—rather than schizotypal—personality traits to be prevalent in a CHR sample, as well as predictive of a transition to psychosis; this psychosis-predictive affect was mainly attributed to deficits in social interaction, rather than indifference and emotional coldness. In our sample, the higher prevalence of schizotypal traits is not surprising, since schizotypal PD is linked with psychotic disorder, both phenomenologically (i.e., both disorders involve positive and negative psychotic-like features) and physiologically (i.e., both disorders are associated with similar genetic and neurobiological factors) (42, 43). Moreover, in line with previous studies and meta-analyses (12, 16, 18), CHR adolescents in our study showed pervasive and more clinically relevant borderline and avoidant traits, as well as poorer adaptive functioning, relative to adolescent clinical groups. These findings suggest that the emotional dysregulation, dissociative experiences, transient paranoid ideation, and psychosis-like symptoms that are included in borderline personality pathology, as well as the avoidant personality traits of increased sensitivity to interpersonal relationships and high levels of anxiety, could partially explain the CHR clinical morbidity.

Of note, the co-occurrence of the CHR state and schizotypal and borderline PDs is questionable from a diagnostic and conceptual standpoint, as it is complicated by a phenomenological overlap. In the first half of the 20th century, schizotypal and borderline PD criteria were developed to provide more reliable descriptors of the so-called “borderline” or “latent schizophrenia” states—meant to indicate characteristics, traits, and symptoms indicative of schizophrenia liability [(44, 45); for a review, see also (43, 46)]. These historical vicissitudes regarding the diagnostic boundaries between certain PDs and psychosis spectrum disorders has led to “conceptual circularity,” impacting research on the relationship between personality traits, PDs, and CHR status. The empirically derived prototypic description of CHR personality characteristics outlined in the present study could overcome this limitation, as it extends beyond the current nosology of PDs, simply describing the observations of clinicians in daily practice.

The multifaceted and complex portrait obtained in the present study provides valuable information on broad aspects of the psychological functioning of CHR individuals. Looking at this picture as a Gestalt, it seems to tap into different dimensions of the schizotypy construct (47). The schizotypy construct refers to the continuum of positive, negative, and disorganized psychotic-like signs and symptoms, ranging from healthy to pathological, that has been theoretically considered—and empirically demonstrated—to predict schizophrenia-spectrum disorders (48–50). In particular, odd thinking and behaviors, unusual perceptual experiences, and suspiciousness could refer to positive symptoms of schizotypy, which are included in the UHR criteria. In fact, the UHR criteria^2^ mainly pertain to sub-threshold psychotic-like experiences, as defined by Chapman and Chapman (51), as well as positive features of schizotypy (50, 52). On the other hand, symptoms of avolition, abulia, blunted affect, and impaired role and academic/occupational functioning account for the negative dimensions of schizotypy. It is important to note that the present study produced no findings for the negative symptom of asociality, which refers to reduced social initiative due to decreased interest in establishing close relationships with others (53–55). In the SWAP-A, asociality is assessed by the item “Appears to have little need for human company or contact; is genuinely indifferent to the presence of others” and, in purely behavioral terms, by the item “Lacks close friendships and relationships.” Interestingly, neither of the abovementioned items was included in our prototypic description of the CHR personality. On the contrary, this description included a relatively high number of SWAP items referring to interpersonal relationships characterized by social anxiety, avoidance of social interaction, and fear of rejection (e.g., item 60, “Tends to be shy or self-conscious in social situations”; item 124, “Tends to avoid, or try to avoid, social situations because of fear of embarrassment or humiliation”; item 86, “Tends to feel ashamed or embarrassed”; item 98, “Tends to fear s/he will be rejected or abandoned by those who are emotionally significant”). Therefore, our results unexpectedly point to avoidant interpersonal strategies, rather than asociality, in the CHR population. It appears that CHR individuals preserve the motivation for social contact but avoid social situations due to feelings of shame or embarrassment, or fear of embarrassment, humiliation, and rejection. Avoidance of social interactions could also be explained by an incapacity to properly cope with the salience of both social and physical stimuli (56, 57), which might be perceived as overwhelming. Such an experience might lead to a general inhibition that diminishes expression in interpersonal contexts (57). Deficits in social functioning in CHR individuals represent a relatively underresearched area, partly due to the high variety of research constructs involved. For example, the construct of interpersonal sensitivity describes a personality trait characterized by “an undue and excessive awareness of, and sensitivity to, the behavior and feelings of others. particularly to perceived or actual situations of criticism or rejection.” [p. 342 (58)]; it has been found to be heightened in CHR individuals, compared to those who have screened negative to psychosis risk (59, 60). Interpersonal sensitivity has also been shown to be associated with difficulties in mentalization (61), represented by a diminished capacity to understand one's own and others' behavior and intentions, thereby hindering proper interpersonal communication and leading to interpersonal withdrawal (62).

Our results also point to significant indicators of impaired social cognition in the CHR sample (i.e., “Has difficulty making sense of other people's behavior; often misunderstands, misinterprets, or is confused by others' actions and reactions”; “Has little psychological insight into own motives, behavior”) (63). To date, mentalizing difficulties in CHR individuals have been primarily investigated in terms of neurocognition, using theory of mind (ToM; i.e., the ability to infer the mental states of others) tasks to demonstrate significant moderate deficits in affect recognition and discrimination of faces, voices, and verbal ToM (64). Moreover, recent findings have also shown that impaired mentalization [as assessed by the Reflective Functioning Scale (RFS) (65)—a quantified index of mentalization ability that is applied to clinical interview transcripts] is more severe in CHR individuals compared to help-seeking clinical controls, strongly associated with APS (SIPS scales), and a significant predictor of the transition to psychosis (66).

Major impairments in social functioning and mentalization could also be attributed to the (less considered) disorganized dimensions of schizotypy (67). These dimensions refer to both cognitive and emotional dysregulation (67, 68), including symptoms such as odd speech and behavior, as well as unusual thought processes and intense emotional experiences that are difficult to mentalize (49, 67). The current study found specific indicators of difficulties in emotional regulation (i.e., “Emotions tend to spiral out of control, leading to extremes of anxiety, sadness, rage, etc.”; “Is unable to soothe or comfort him/herself without the help of another person [i.e., has difficulty regulating own emotions]”), in line with phenomenological accounts of the role of emotional dysregulation prior to the onset of psychosis (69). Our group comparisons also revealed that the CHR sample showed higher borderline personality traits (marked by emotional dysregulation that severely affects global functioning and interpersonal relationships) than the clinical group without PDs. Such findings speculatively link the positive and disorganized dimensions of schizotypy through cognitive dysfunction in the ability to properly deal with stress (68).

Symptoms of emotional instability or borderline personality traits may also be signified in terms of a Bleulerian *ambivalence* (70). Considering the lack of self-insight and self-consciousness that is frequently presented by CHR adolescents (71–73), it is reasonable to suppose that CHR youths may perceive several emotions simultaneously and that this could be a chaotic and overwhelming experience that they are unable to elaborate through higher-order cognition. In this perspective, the constellation of psychological symptoms in the empirical prototype presented here (i.e., avoidant interpersonal strategies, impaired mentalization, difficulties in emotional regulation) could be understood as the result of a lack of integration between emotions and cognitions—also derived from the atypical brain development observed in CHR individuals and those on the schizophrenia-spectrum (74).

Overall, the CHR personality prototype derived in the present study can reveal important targets for psychosocial interventions. For example, mentalization-based treatments (75, 76) have been shown to be effective in reducing social anxiety and promoting more adaptive emotional strategies (77), as well as in enhancing mentalizing (78). In a similar vein, the new group of therapies referred to as the “third wave” (79) of behavioral and cognitive therapies [e.g., dialectical behavior therapy (80), functional analytic therapy (81), integrative behavioral couples therapy (82), acceptance and commitment therapy (83), and mindfulness-based cognitive therapy (84)] might meet the clinical needs of CHR youths (85) by focusing on contextual and experiential change strategies, including acceptance, cognitive defusion, mindfulness, relationships, values, emotional deepening, contact with the present moment, and related ideas (86).

Some limitations of the present study should be noted and discussed. First, the cross-sectional nature of the research did not allow us to examine the role of personality in clinical outcomes over the long term. In particular, future studies should seek to establish whether specific personality traits and/or disorders may adversely affect or moderate the outcomes of preventive treatments in the CHR population^3^. Second, the SWAP-200-A data were produced by different clinical centers, and raters were not equally distributed across the three groups of patients. Consequently, effects reflecting rater assessment differences (i.e., rater bias) cannot be completely excluded. However, we assume that any rater bias, if present, would be trivial, since all participating clinicians were trained to administer the SWAP-200-A assessment and obtained an IRR in the range of 0.69–0.75. Third, the SIPS was not administered to the control groups to rule out CHR status in these individuals; this may have affected the validity of the grouping variable. Nevertheless, as specified, in all recruitment sites for non-CHR patients, specific exclusion criteria were applied to overcome this limitation (see “Methods” section). Moreover, the validity of the UHR criteria strongly depends on the specific population to which they are applied. Specifically, there is compelling evidence that the UHR criteria lack validity when the criteria are applied to so-called “unselected psychiatric samples” (i.e., individuals who have not been referred to a clinical service specializing in the early detection and treatment of psychosis), especially in the younger population (33, 34, 88)–as was the case in the present study. Finally, although the SWAP-200-A assessment provides a broad and deep evaluation of psychological functioning, the literature on psychosis predominantly includes diagnostic approaches emphasizing the distress that is subjectively experienced by patients [e.g., basic symptoms (89) and minimal self-disturbances (90)], rather than signs and symptoms that are detectable by external observers. This leads to a paradox in which the same assessment method can reliably measure some key phenomenological elements of psychotic-spectrum disorders but fail to reliably assess other clinical aspects that might be crucial for treatment planning (e.g., personality).

## Data Availability Statement

The data of this study are not available due to ethical concerns. We must protect patient privacy and security and follow the ethical rules of our institutions and their restrictions on data sharing.

## Ethics Statement

The study obtained the approvals of Ethics Committee of the Bambino Gesù Clinical and Research Hospital and the Ethics Committee of the Department of Dynamic and Clinical Psychology of Sapienza University of Rome (n°44/2017). All clinicians furnished written informed consent and were instructed to withhold any identifying information about their patients. They received no remuneration for their participation. Adolescent patients were not directly involved in this study.

## Author Contributions

TB conceived the research study and wrote the first draft of the manuscript. AT conceived the research study and contributed to data analysis/interpretation and the writing of the manuscript. GDC contributed to the writing of the manuscript. MT and IG collected data. SS, SV, and VL contributed to the interpretation of the results and critically reviewed the final draft of the manuscript. MP assisted with data collection and contributed to the study design. All authors contributed to the article and approved the submitted version.

## Conflict of Interest

The authors declare that the research was conducted in the absence of any commercial or financial relationships that could be construed as a potential conflict of interest. The handling editor declared a past collaboration with one of the authors SV.

## Footnotes

^1^It would seem that the presence of a PD does not affect clinical outcomes, irrespective of a transition to full-blown psychotic disorder. For example, a recent investigation by Polari et al. (15) demonstrated that an additional diagnosis of borderline personality disorder was not associated with poorer outcomes, in terms of the recurrence, relapse, and remission of APS, as well as general functioning. However, further research is needed, and these results should also be replicated for outcomes other than APS.

^2^However, it is important to note that the schizotypy and UHR criteria refer to complementary but different aspects of vulnerability to psychosis: the former refer to *trait* indicators of vulnerability (i.e., lifelong temporal stability), whereas the latter mainly focus on *state* signs of an imminent transition to full-blown psychotic disorder (48, 50).

^3^A variability ratio meta-analysis (87) found no evidence for differences in individual responses to preventive treatments in CHR individuals; nevertheless, no outcomes other than APS were explored, and it was impossible to exclude the possibility that *subsets* of CHR individuals may have systematically responded differently to preventive treatments.
